# Genome-wide association study and haplotype characterization of bacterial kidney disease (BKD) resistance in Atlantic salmon (*Salmo salar*) across two generations

**DOI:** 10.3389/fgene.2026.1985591

**Published:** 2026-10-28

**Authors:** Angela Astudillo, Ross Houston, Jean Paul Lhorente, Baltasar Garcia

**Affiliations:** 1 Benchmark Genetics, Puerto Varas, Chile; 2 Benchmark Genetics, Bergen, Norway

**Keywords:** Aquaculture genetics, BKD, GWAS, *Salmo salar*, selective breeding

## Abstract

**Introduction:**

Bacterial Kidney Disease (BKD) is a disease affecting Atlantic salmon aquaculture. This study aimed to evaluate the genetic architecture of resistance to BKD across two generations of the same genetic line in an Atlantic salmon (Salmo salar) breeding program, characterize favorable haplotypes associated with resistance, and identify candidate genes related to immune response to support future selective breeding strategies.

**Methods:**

Genome-wide association analyses were conducted for mortality and time-to-death traits across two generations. Heritability was estimated, and haplotype analysis was performed to characterize favorable haplotypes associated with resistance. Candidate genes were proposed based on their immune relevance and proximity to significant SNPs.

**Results:**

Genome-wide association analyses identified a significant region on chromosome 25 containing 36 significant single nucleotide polymorphisms (SNPs), with p-values as low as 5.94 × 10^-36^. Heritability estimates ranged from low to moderate, from 0.147 ± 0.01 to 0.197 ± 0.01 on average. Haplotype analysis identified a two-SNP haplotype associated with an average reduction in mortality of 17.45% and an increase of approximately 3 days in survival time under challenge conditions. Seven candidate genes were proposed for further validation.

**Discussion:**

These findings suggest that genomic selection should remain the primary strategy for improving BKD resistance, while marker-assisted selection targeting the identified chromosome 25 region may provide complementary value by capturing a moderate but biologically relevant proportion of the genetic variation associated with disease resistance in Atlantic salmon.

## 1 Introduction

The rise of genetic studies and breeding programs for the aquaculture industry has highlighted the need to obtain a high-quality product in the smallest amount of time, but also to ensure that the changes keep improving in future generations. Through selective breeding, populations can be improved cumulatively, with each generation showing progress in traits such as growth, disease resistance, fillet quality and other economically important traits. Selective breeding can accelerate genetic improvement and also be used as a tool to reduce the risk of inbreeding and other undesirable effects associated with conventional breeding practices. In the Atlantic salmon (*Salmo salar*) industry, a large portion of losses is related to the emergence of diseases. For this reason, naturally increasing the disease resistance of populations is a crucial objective for a sustainable genetic program in modern aquaculture (Gjedrem, 2012; Yáñez et al., 2014). Specifically, for Atlantic salmon (*S. salar*), diseases such as sea lice (*Caligus rogercresseyi*), *Piscirickettsia salmonis* (SRS), and infectious salmon anemia (ISA) have been prioritized for the improvement of resistance due to the severe impact on production performance worldwide (Robledo et al., 2018a; Li et al., 2011; Correa et al., 2015; Robledo et al., 2019). In a traditional breeding program, an important step is to estimate trait heritability using pedigree information. This provides an indication of the proportion of phenotypic variation explained by additive genetic effects and, consequently, the expected potential response to selection. With advances in genomic technologies, it is now possible to include DNA information in genetic evaluations. This has been largely enabled by the identification and use of single nucleotide polymorphisms (SNPs), which provide genome-wide markers to capture genetic variation among individuals. To better understand the genetic basis of disease resistance, Genome-wide association studies (GWAS) have been used in breeding programs to describe the genomic architecture of a trait by testing for statistical associations between phenotypic variation and SNP markers across the genome. When sufficient linkage disequilibrium exists between SNPs and quantitative trait loci (QTLs), these associations can help identify genomic regions linked to the trait and, in some cases, suggest potential causal variants (Goddard and Hayes, 2009). GWAS have been applied to several relevant diseases in Atlantic salmon, such as the ectoparasite sea lice (Correa et al., 2017; Cáceres et al., 2024), SRS bacterial disease (Marín-Nahuelpi et al., 2024), pancreas disease (Hillestad et al., 2020b), Amoebic Gill disease (Robledo et al., 2018b; Aslam et al., 2020), and Cardiomyopathy syndrome (Hillestad et al., 2020a). The genetic architecture of disease-resistance traits can vary. In some cases, resistance traits are controlled by one or a few genomic regions with large effects, known as monogenic or oligogenic. For Atlantic salmon, some examples are Infectious Pancreatic Necrosis (IPN) (Houston et al., 2008) and Cardiomyopathy Syndrome (CMS) (Hillestad and Moghadam, 2019). These are favorable cases for marker-assisted selection (MAS), as selection can target the specific loci for disease resistance. However, most of the studied traits have a polygenic architecture, meaning that multiple small loci contribute to the observed variation, such as for SRS and sea lice infection (Cáceres et al., 2024; Marín-Nahuelpi et al., 2024). For this genomic architecture, it is recommended the implementation of genomic selection rather than a MAS approach. Therefore, GWAS are important for selective breeding, not only for finding candidate regions, but also for determining breeding strategies for the desired trait.

Among the major diseases that affect Atlantic salmon aquaculture, the Renibacteriosis, also known as Bacterial kidney disease (BKD), is one of the most important disease in most cold water of Chilean industry. BKD is caused by the Gram-positive bacterium *Renibacterium salmoninarum* and it is a problem for Atlantic salmon aquaculture worldwide, mostly prevalent in the cold northern waters of the Atlantic and Pacific Oceans (Burnley et al., 2010; Wiens, 2011). The disease is chronic in nature and presents symptoms such as kidney nodular lesions, lethargy, skin darkening, and hemorrhages (Wiens, 2011; Evenden et al., 1993; Bruno, 1986). It is transmissible both horizontally, from fish that share the same environment, and vertically, where the bacteria is passed down to the eggs (Wiens, 2011; Bruno, 1986; Burnley et al., 2010). BKD leads to increase in mortality, reduced overall fish health, and growth, resulting in production losses and higher costs associated with disease control (Burnley et al., 2010; Evenden et al., 1993; Wiens, 2011). In Chile, mortality associated with BKD accounted for 4.5% of total secondary mortality in 2024, while in the Magallanes region specifically, BKD represented 26% of the diagnoses reported for marine farms (Sernapesca, 2025). According to a previous study, in a European Atlantic salmon population, the heritability values for binary mortality (dead or alive) were estimated between 0.23 ± 0.10 and 0.27 ± 0.08 using different statistical models with pedigree information only (Gjedrem and Gjøen, 1995). For a North American population and using 50K SNP panel, another study reported heritability estimations of binary mortality and time to death of 0.143–0.145, respectively (Holborn et al., 2018).These estimates fall within the low to moderate range, indicating that selective breeding could lead to genetic improvement in these populations. In addition to estimating genetic parameters (Holborn et al., 2018), also investigated the genetic architecture of BKD resistance using binary mortality and time to death as target traits. The authors found no evidence of major-effect loci, suggesting that resistance to BKD is likely controlled by a polygenic genetic architecture. However, these conclusions should be interpreted with caution, as the results are closely linked to the study design. In particular, the use of a Canadian breeding population, the SNP array density, and the population structure may directly influence the inferred genetic architecture. Since this remains the only genome-wide study of BKD resistance in Atlantic salmon reported to date, further studies are needed to validate these findings across different populations, genetic backgrounds, and genotyping platforms.

In this context, this study aims to evaluate BKD resistance across two generations of the same genetic line in an Atlantic salmon (*S. salar*) breeding program by performing GWAS analyses for two disease-resistance traits, identifying significant SNPs, characterizing favorable haplotypes associated with resistance, and proposing candidate genes related to immune response within genomic regions explaining the largest proportion of phenotypic variance, with the goal of supporting future breeding selection strategies.

## 2 Materials and methods

### 2.1 Study population and BKD challenge

The fish population used in this study belongs to the SAGA strain, which originated from the Icelandic Benchmark Genetics breeding program and was subsequently transferred to Chile in 2017 to establish the Atlantic salmon breeding program. The program integrates pedigree and genomic information into a selection index targeting resistance to important diseases, flesh quality and colour, growth, and carcass traits. Two year classes from the same genetic line were evaluated: YC18 (2,954 fish from 180 families; mean initial body weight 54.8±11.3 g) and YC22 (2,210 fish from 190 families; mean initial body weight 89.3±18.9 g). Fish in both year classes were pre-smolts.

Challenges were conducted at a specialised fish-pathogen experimental facility under its established animal-welfare procedures. YC18 fish were acclimatised for 5 days and then allocated among four 2 ${\text{m}}^{3}$ freshwater challenge tanks, with similar mean body weight and equal numbers of fish per tank (approximately 16 fish per family). Initial biomass density was 20–25 kg ${\text{m}}^{-3}$ . During the challenge, water temperature was 10.2±0.3 °C , oxygen saturation was 100.9±3.8% , the photoperiod was 24 h light, and water exchange was 1.2–1.5 tank volumes ${\text{h}}^{-1}$ . Fish were fed a commercial diet automatically at an Specific Feeding Rate (SFR) of 0.5%–1.0% and fasted for 48 h before inoculation.

YC22 fish were acclimatised for 17 days in two freshwater tanks before being pooled into one 15 ${\text{m}}^{3}$ freshwater tank for the challenge (12 fish per family). Family allocation and initial body weight were recorded before transfer to the experimental facility. Initial biomass density in the challenge tank was 16.0 kg ${\text{m}}^{-3}$ . Water quality was monitored throughout acclimation and challenge, with dissolved oxygen recorded using the facility’s oxygen-saturation monitoring record (TB-RE-19). During acclimation, mean water temperature and dissolved oxygen were 11.3±0.38°C and 8.8±0.44 mg ${\text{L}}^{-1}$ , respectively. During the challenge, mean water temperature and dissolved oxygen were 10.7±0.20°C and 8.7±0.59 mg ${\text{L}}^{-1}$ , respectively. Fish were fed manually with a 3-mm commercial diet at an SFR of 0.94±0.32% and fasted for 48 h before inoculation.

In both year classes, fish were anaesthetised with 20% benzocaine to deep anaesthesia (plane III) before intraperitoneal inoculation with *R. salmoninarum* isolate AY-8009 (supplied as a bacteriological culture by ADL Diagnostic, Puerto Montt, Chile). Intraperitoneal inoculation was selected to standardise pathogen dose and exposure time across fish, thereby reducing variation in pathogen exposure and improving power to detect family-level differences in resistance. This model bypasses natural mucosal exposure and should therefore be interpreted as systemic resistance under controlled challenge conditions (Gudmundsdóttir et al., 2017). The inoculum was administered at 0.1 mL ${\text{fish}}^{-1}$ , using a 1:100 dilution selected in a previous ${\text{LD}}_{50}$ dose-finding study. Inoculum concentrations were $6.4\times 10^{8}$ CFU ${\text{mL}}^{-1}$ in YC18 and $1.0\times 10^{9}$ CFU ${\text{mL}}^{-1}$ in YC22. No uninfected control group was included; therefore, mortality and time-to-death should be interpreted as measures of relative BKD resistance among fish exposed to the same standardized challenge. Nevertheless, diagnostic assessment of mortalities was performed during both challenges to confirm findings consistent with BKD. Fish were monitored for 60 days in YC18 and 56 days in YC22, and mortalities were removed and recorded daily.

Each fish was assigned a correlative identification code linked to its individual PIT tag. For each mortality, date of death, body weight at death, binary mortality status, sex, tank, and macroscopic necropsy findings were recorded. Caudal-fin samples were collected from all challenged fish and stored in ethanol for subsequent genotypic analyses. For diagnostic assessment, 15 mortalities per tank in YC18 (60 fish in total) were submitted for necropsy and BKD RT-PCR. In YC22, all mortalities underwent necropsy, and anterior-kidney samples were collected from 100 mortalities at different stages of the mortality curve.

At the end of each challenge, surviving fish were euthanised by anaesthetic overdose. Final body weight, sex, survival status, PIT tag, and caudal-fin samples were recorded. All fish were disposed of through the facility’s authorized silage-disposal system. Challenge procedures followed the internal animal-welfare procedures of the experimental facility and the ethical-approval waiver applicable under Chilean Law No. 20.380.

### 2.2 Genotyping

After the end of the challenge, fin samples were submitted to the IdentiGEN Genotyping Laboratory for DNA extraction and genotyping with the commercially available Benchmark Genetics 70K Affymetrix Axiom SNP array (https://www.bmkgenetics.com/services/genotyping-services/), mapped to the Atlantic salmon reference genome assembly (Ssal_v3.1 (Norwegian University of Life Sciences, 2021); NCBI GenBank accession GCA_905237065.2). Single nucleotide polymorphism (SNP) quality control (QC) was evaluated using standard filtering criteria, using Plink software version v1.9.0-b.7.8 64-bit (15 June 2025) (Chang et al., 2015), with genotype call rate (GENO) of 0.1, minor allele frequency (MAF) of 0.05, Hardy–Weinberg equilibrium p-value threshold with Bonferroni correction according the total SNP count, and individual missingness (MIND) of 0.1. Quality control procedures were applied separately to each generation. Following QC, datasets were merged and all downstream analyses were performed according to three groups: YC18, YC22 and the combined YC18_YC22 dataset.

### 2.3 Genetic parameter estimation

The heritability $\left(h^{2}\right)$ of each trait was estimated using the linear mixed model defined above, incorporating a genomic relationship matrix (GRM) to account for the additive genetic variance (Equation 1). Variance components were estimated with a restricted maximum likelihood (REML) with an average information algorithm, and SNP-based heritability was calculated as:

$$h^{2}=\frac{\sigma_{g}^{2}}{\sigma_{g}^{2}+\sigma_{e}^{2}}$$ (1)

where $\sigma_{g}^{2}$ is the additive genetic variance captured by the GRM, and $\sigma_{e}^{2}$ is the residual variance.

GWASs were performed using a linear mixed model (LMM) applied through the software GCTA (Yang et al., 2011a), to account for population structure and relatedness (Equation 2). The model for each trait was:

$$y=X\beta +Zg+x_{j}b_{j}+\varepsilon$$ (2)

where:

-
y is the vector of phenotypes (Time of death and binary mortality).
-
Xβ represents fixed effects including covariates (sex, initial weight and year-class).
-
Zg is the random polygenic effect captured by the genomic relationship matrix (GRM).
-
$x_{j}$ is the genotype vector for SNP j and $b_{j}$ is its effect
-
ε is the residual error.

Sex and year class were fitted as categorical fixed effects, while initial body weight was included as a continuous covariate in the GWAS and variance-component models. SNP effects were estimated using the MLMA–LOCO approach implemented in GCTA where each SNP is tested while excluding its corresponding chromosome from the genomic relationship matrix to avoid proximal contamination (Yang et al., 2014). Genome-wide significance was assessed using a Bonferroni correction based on the total SNP count, applying a threshold of 0.05/ Total SNPs. Chromosome-wide significance was determined using a chromosome-specific Bonferroni correction assuming equal SNP distribution across 29 chromosomes, resulting in a threshold of 0.05/(totalSNPs/29) .

### 2.4 Genomic block description and haplotype analysis

Genomic blocks containing significant SNPs were defined using a physical clustering strategy combined with linkage disequilibrium (LD) characterization in the combined YC18_YC22 dataset.

First, genome-wide significant SNPs were grouped into genomic blocks using a physical distance threshold of 0.5 Mb, where consecutive significant SNPs separated by less than 0.5 Mb were assigned to the same block. Within each block, the lead SNP was defined as the marker with the lowest *p*-value. For each trait, all significant SNPs assigned to the same genomic block were jointly fitted in a multiple linear regression model to estimate the variance explained by that region.

To quantify the contribution of each genomic region to trait variation, the proportion of phenotypic variance explained by significant genomic regions was estimated using multi-SNP conditional linear models (Equation 3) (Yang et al., 2011b; 2010). For each trait, binary mortality and time of death, all significant SNPs located within the same genomic window (0.5 Mb) were jointly fitted in a multiple linear regression model to estimate the variance explained by that region:

$$Y=\mu +\overset{k}{\underset{i=1}{\sum }}\beta_{i}X_{i}+\varepsilon$$ (3)

where Y is the phenotype, $X_{i}$ is the genotype of the i -th SNP in the window, $\beta_{i}$ is its regression coefficient, and k is the number of SNPs in the window.

In parallel, the LD structure of each genomic block was characterized by calculating the maximum pairwise $r^{2}$ between the lead SNP and all other markers located on the same chromosome using PLINK. This metric was used to describe the LD strength of the association signal, rather than to define fixed LD-based block boundaries.

The variance explained by each window was calculated as the adjusted coefficient of determination $\left(R_{\text{adj}}^{2}\right)$ (Equation 4) extracted from each fitted model and expressed as a percentage to obtain the adjusted window-level phenotypic variance explained (Adjusted PVE):

$${\text{PVE}}_{\text{window}}=R_{\text{adj}}^{2}\times 100$$ (4)

Adjusted PVE values were reported separately for each trait to account for model complexity and to prevent inflation due to the inclusion of multiple correlated markers within a region.

Finally, for each genomic block, genetic variance explained (GVE) was approximated by scaling the adjusted phenotypic variance explained by the trait heritability (Equation 5):

$${GVE}_{\text{block}}=\frac{{PVE}_{\text{block}}}{h^{2}}$$ (5)

where ${PVE}_{\text{block}}$ is the adjusted phenotypic variance explained by the genomic block, and $h^{2}$ is the SNP-based heritability estimated from the genomic relationship matrix.

For the haplotype-based evaluation, SNPs associated with resistance-related traits were selected based on genome-wide association analyses. A multi-locus genotype approach was applied, in which combinations of alleles across the selected SNPs were used to define haplotype classes. Individual fish were grouped according to their multilocus allelic combinations, and the phenotypic values for each BKD-related trait were averaged within each group. To ensure robustness of the estimates, only haplotype groups represented by a minimum of ten individuals were retained for subsequent analyses (Stram, 2005).

Statistically, mortality was analyzed using a binomial generalized linear model because it was recorded as a binary trait, whereas time to death, treated as a continuous variable, was analyzed using a linear model. Consequently, global haplotype effects were evaluated using a likelihood ratio $\chi^{2}$ test for mortality and an ANOVA F-test for time to death, from which the reported p-values were derived.

To further evaluate whether haplotype effects were consistent within shared genetic backgrounds, a linear mixed-effects model was fitted as:

$$M_{\mathrm{ijk}}=\mu +H_{i}+\left(1|F_{j}\right)+\varepsilon_{\mathrm{ijk}},$$ (6)

where $M_{\mathrm{ijk}}$ is the mortality phenotype, $H_{i}$ is the fixed effect of haplotype class, and $\left(1|F_{j}\right)$ represents a random intercept for family. Significance of the haplotype effect was assessed using a t-test on the fixed-effect estimate, from which corresponding t-values and p-values were derived. The resulting genomic blocks were subsequently used for candidate gene identification.

### 2.5 Candidate genes identification

SNP positions were annotated according to the Atlantic salmon reference genome used for the genomic analyses. Based on the overall impact of each genetic block on resistance traits, windows explaining >1% adjusted PVE for at least one trait were chosen. This threshold was used as a transparent and inclusive prioritisation criterion for biological interpretation, rather than as evidence of a large-effect QTL or a statistical significance threshold. Candidate genes were then investigated according to the described interval. From the resulting gene set, those with previously reported involvement in immune-related processes were selected as candidates. Functional relevance was assessed through a targeted literature review using web search. Searches were performed using combinations of the gene name with keywords including “immunity,” “fish,” “Atlantic salmon,” and “bacterial infection.”

## 3 Results

From the challenge experiments, we obtained 5,074 fin samples and corresponding individual phenotypic records. Sex ratios were broadly balanced across year classes. YC18 comprised 45.2% females and 54.8% males, while YC22 comprised 49.5% females and 50.5% males. Across both challenges, mean mortality was 72.4% ± 0.01 and the mean time to death was 49.2 ± 0.11 days. Macroscopic findings in mortalities were compatible with BKD and included pale gills, splenomegaly, hepatic nodules, hepatomegaly, renomegaly, and pseudomembranes on internal organs.

Sex ratios were broadly balanced across year classes. YC18 comprised 45.2% females and 54.8% males, while YC22 comprised 49.5% females and 50.5% males. Sex was not significantly associated with mortality (p=0.391) or time to death (p=0.979) . Males showed greater adjusted weight change than females ( +3.12 g, p=0.001 ), although this effect differed between year classes (sex × year class, p=0.018 ). Sex was retained in the model because it improved model fit (lower −2 log-likelihood) and yielded heritability estimates closer to previously reported values for BKD resistance.

Phenotypic variation was observed both within and between year classes (YC). Survival patterns differed between generations: YC18 exhibited lower mortality, while YC22 showed a greater weight difference (Table 1). These contrasts provide the phenotypic variance required for subsequent quantitative genetic analyses.

**TABLE 1:** Mean ( ± SD) weight change, binary mortality (Mortality), and time to death (BKD Time) for two Atlantic salmon year classes following experimental challenge for BKD.

YC	N	Weight change (g)	Mortality (%)	BKD time (days)
2018	2,864	+9.24 ± 0.43	0.682 ± 0.009	50.78 ± 0.16
2022	2,210	+28.74 ± 1.10	0.777 ± 0.009	47.16 ± 0.15
All	5,074	+17.73 ± 0.55	0.724 ± 0.006	49.20 ± 0.11

Weight change was calculated as final minus initial body weight; mortality is a binary trait (0 = alive, 1 = dead), shown as percentage; BKD, Time indicates days to death post-challenge.

### 3.1 SNP QC results

From the genotyped samples, quality control filtering was applied independently to YC18 and YC22 datasets. After quality control, the filtered datasets were merged based on shared markers, resulting in a combined dataset containing 47,816 SNPs across 4,846 individuals (Table 2). The reduction in marker number relative to the independently filtered datasets was primarily due to differences between array versions, particularly in SNP positioning, resulting in the loss of approximately 22%–25% of markers during the merging process.

**TABLE 2:** Summary of genotype quality control filtering applied independently to each Atlantic salmon year class.

Dataset	Initial SNPs	After Geno	After MAF	After HWE	Final SNPs	Final samples
YC18	69,411	68,049	64,892	61,386	61,386	2,678
YC22	68,497	68,282	65,146	63,526	63,526	2,168

GENO, maximum proportion of missing genotypes allowed per SNP (0.10); MAF, minor allele frequency (0.05); HWE, Hardy–Weinberg equilibrium test threshold adjusted using Bonferroni correction.

### 3.2 Heritability and GWAS results

When analysed separately by generation, heritability estimates for BKD resistance traits were low to moderate in YC18 and moderate in YC22, indicating additive genetic variation within both year classes (Table 3). In YC18, heritability was estimated at 0.147±0.02 for binary mortality and 0.185±0.02 for time to death. In YC22, estimates were classified as moderate, at 0.220±0.03 for binary mortality and 0.297±0.03 for time to death. Across both year classes, time to death showed higher heritability than binary mortality, suggesting that it may capture additional genetic variation related to BKD resistance.

**TABLE 3:** Variance components and heritability estimates for BKD resistance traits across year classes and overall estimates.

Trait	Component	YC18	YC22	Overall
Mortality	V(G)	0.0317±0.01	0.0373±0.01	0.0285±0.00
	V(e)	0.1835±0.01	0.1324±0.00	0.1660±0.00
	$V_{p}$	0.2153±0.01	0.1698±0.01	0.1945±0.00
	$h^{2}$	0.147±0.02	0.220±0.03	0.147±0.01
Time of death	V(G)	12.53±1.85	13.66±1.47	11.46±0.94
	V(e)	55.37±1.95	32.31±1.20	46.45±1.04
	$V_{p}$	67.90±2.13	45.96±1.57	57.91±1.29
	$h^{2}$	0.185±0.02	0.297±0.03	0.197±0.01

V(G) = additive genetic variance; V(e) = residual variance; $V_{p}$ = phenotypic variance; $h^{2}$ = narrow-sense heritability $\left(V\left(G\right)/V_{p}\right)$ . Values are presented as estimate ± standard error. YC18 and YC22 correspond to separate year-class analyses, while Overall refers to the full dataset. Heritability values are bolded.

Genome-wide association analysis identified multiple SNPs significantly associated with resistance to bacterial kidney disease (BKD). The strongest association signals were concentrated on chromosome 25, where several SNPs reached higher levels of significance, with p-values as low as $10^{-36}$ in the time to death trait. Importantly, these loci displayed consistent association patterns across both BKD resistance traits, suggesting a shared underlying genetic architecture (Figure 1). The quantile-quantile and Manhattan plots for each year-class are presented separately in Supplementary Figures 1.1 and 1.2, respectively.

**FIGURE 1:**
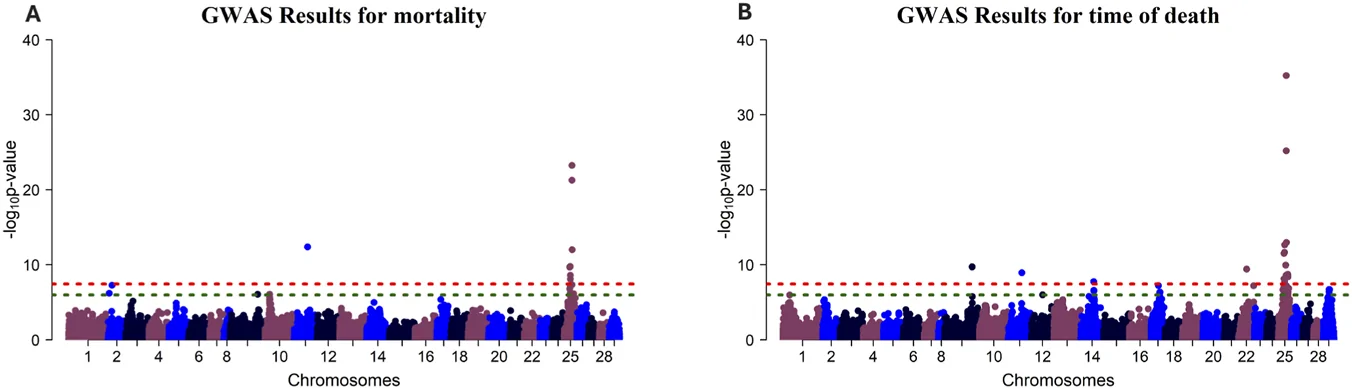
Genome-wide association results for BKD resistance in *Salmo salar* using combined data from both year classes. **(A)** Manhattan plot for mortality. **(B)** Manhattan plot for time to death. The green dashed line denotes the genome-wide Bonferroni significance threshold ( α=0.05/47,816 SNPs). The red dashed line indicates a more stringent threshold additionally accounting for multiple testing across the 29 chromosomes (α=0.05/(47,816×29)) .

The GWAS identified 54 genome-wide significant SNPs associated with BKD resistance traits. Of these, 36 SNPs were located on chromosome 25, indicating that this chromosome contained the main association signal detected in the study. These SNPs were significant for at least one of the evaluated traits, binary mortality or time of death (Supplementary Figure 1.3). Most associations were detected for time of death, suggesting that this trait provided greater power to capture genomic variation related to BKD resistance than binary mortality. There were 13 SNPs shared between both traits (Table 4). The genetic correlation between time to death and binary survival was close to unity, indicating that both traits are largely controlled by the same underlying genetic mechanisms (results not shown).

**TABLE 4:** All 13 unique SNPs on chromosome 25 in *Salmo salar* that exceeded the genome-wide significance threshold in both BKD resistance traits.

SNP	Chr	Pos (bp)	A1/A2	MAF	Mort p-value	Time p-value
AX-88110791	25	31,728,863	C/A	0.438	5.75e-24	5.94e-36
AX-88118415	25	31,824,753	T/C	0.379	5.27e-22	6.37e-26
AX-87164891	25	32,044,678	T/C	0.483	9.96e-13	1.07e-13
AX-98296355	25	25,528,053	A/G	0.310	2.45e-09	2.22e-13
AX-96426425	25	22,289,341	C/T	0.353	1.95e-10	3.12e-12
AX-96172802	25	24,359,232	C/T	0.456	1.66e-10	2.21e-12
AX-96352799	25	31,748,699	T/C	0.213	4.49e-08	2.30e-09
AX-88213524	25	38,944,413	A/G	0.246	7.28e-07	2.89e-09
AX-96130927	25	25,027,279	A/G	0.295	8.00e-09	6.20e-09
AX-96458203	25	22,076,103	C/A	0.310	1.57e-07	7.37e-09
AX-96497525	25	24,450,130	T/G	0.220	6.38e-07	1.07e-08
AX-87465426	25	24,396,219	T/C	0.289	8.45e-08	2.00e-08
AX-96179822	25	31,707,394	C/A	0.134	8.29e-07	5.89e-08

The SNP, column corresponds to the Affymetrix Axiom identifier for each SNP; Pos(bp) indicates the genomic position in base pairs; A1/A2 represent the reference and alternative alleles, respectively; MAF, indicates the minor allele frequency; and Mort p-value and Time p-value correspond to the association significance values for mortality and time to death traits, respectively.

### 3.3 Genomic block architecture and haplotype effects on BKD resistance

The significant SNPs detected on chromosome 25 were organized into genomic blocks to describe the regional architecture of the association signal for BKD resistance. These blocks differed in the number of significant SNPs, LD strength with the lead marker, and the proportion of phenotypic variance explained for each trait (Table 5).

**TABLE 5:** Genomic blocks identified on chromosome 25 associated with BKD resistance traits.

Block	Position (bp)	SNP count	Max $r^{2}$ (chr25)	BKD_MORTALITY		BKD_TIME	
				PVE (%)	GVE (%)	PVE (%)	GVE (%)
Block 9	31,562,357–32,044,678	6	0.782	2.30	15.65	3.31	16.80
Block 3	22,076,103–22,289,341	3	0.357	1.06	7.21	1.34	6.80
Block 4	24,359,232–25,027,279	5	0.378	1.18	8.03	1.26	6.40
Block 10	34,086,405–34,086,405	1	0.921	0.65	4.42	1.01	5.13
Block 11	37,772,413–38,167,816	3	0.228	0.83	5.65	0.99	5.03
Block 7	29,454,134–29,709,421	4	0.537	0.85	5.78	0.97	4.92
Block 6	28,251,241–28,510,871	3	0.332	0.88	5.99	0.84	4.26
Block 2	21,503,369–21,503,369	1	0.224	0.60	4.08	0.70	3.55
Block 12	38,944,413–39,112,658	5	0.990	0.60	4.08	0.66	3.35
Block 8	30,608,135–30,609,053	2	0.848	0.28	1.90	0.55	2.79
Block 1	18,226,525–18,226,525	1	0.968	0.68	4.63	0.54	2.74
Block 5	25,528,053–25,528,053	1	0.505	0.45	3.06	0.33	1.68
Block 13	41,586,870–41,586,870	1	0.646	0.12	0.82	0.18	0.91

BKD_MORTALITY, Binary mortality due to BKD; BKD_TIME, time (days) to death following BKD, challenge; PVE (%) = proportion of phenotypic variance explained; GVE (%) = proportion of genetic variance explained.

Overall, the strongest regional signals were observed for time to death, consistent with the larger number of significant SNPs detected for this trait in the GWAS. In contrast, binary mortality showed a more limited signal across the same regions. The adjusted variance explained by each block indicated that the chromosome 25 association was not driven by a single isolated marker, but rather by clusters of significant SNPs within defined genomic regions. These genomic blocks were further explored to characterize favorable haplotypes associated with improved BKD resistance and to identify candidate genes located within the most relevant regions.

Haplotype analysis was performed using the top three SNPs within Block 9, which exhibited the highest PVE across both BKD resistance traits. As shown in Figure 2, a clear pattern was observed, where the SNP1 AA and SNP2 CC combination was associated with lower mortality and higher time to death. This haplotype-based approach allowed the assessment of the cumulative effect of favorable and unfavorable alleles on disease resistance beyond the contribution of individual SNPs (see Supplementary Table 1.4).

**FIGURE 2:**
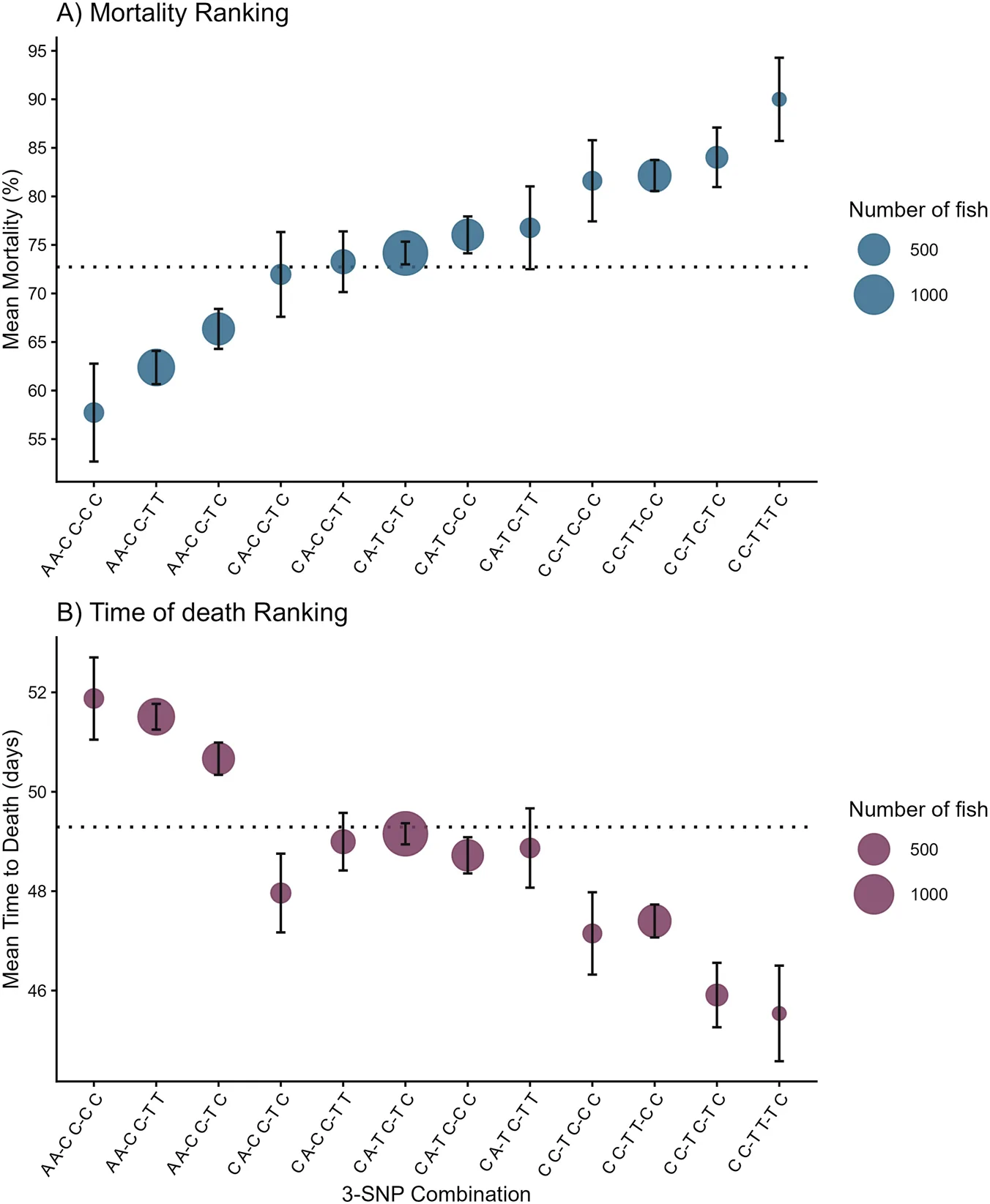
Association between three-SNP haplotype combinations identified by GWAS and BKD resistance traits. **(A)** Mean mortality (%) ranked by haplotype combination. **(B)** Mean time to death (days), displayed in the same order as the mortality ranking for direct comparison. Differences among haplotypes reflect the cumulative effect of allelic combinations on disease resistance. Notably, haplotypes beginning with AA-CC form a distinct group characterized by lower mortality and longer average time to death.

Haplotype groups were visualized using bubble plots, where bubble size reflected the number of individuals per haplotype, enabling evaluation of the distribution and cumulative effects of allele combinations on BKD resistance traits.

Following statistical tests of a binomial generalized linear model ( $\chi^{2}$ test; $p=5.58\times 10^{-20}$ ) for mortality and an ANOVA F-test from a linear model for time to death $\left(p=2.07\times 10^{-31}\right)$ , the haplotype class defined by the *AA-CC* allele combination consistently formed a statistically distinct group characterized by significantly lower mortality and longer mean time to death compared to haplotypes carrying alternative allele combinations. These results were further supported by a linear mixed-effects model including family as a random effect, where the haplotype fixed effect remained highly significant ( t=6.05 , p<0.001 ).

The haplotype analysis evidenced a group associated with a reduced susceptibility to BKD, where two key SNPs (AX-88110791 and AX-88118415), when presented with an AACC pattern, were associated with an average decrease in mortality of 17.45% and an increase of approximately 3 days in time to death (5.6% increase in survival time). These patterns were also consistent when the analysis was performed within year classes, with mortality reductions of 17.8% in YC18% and 16.2% in YC22, together with an approximate increase of 3 days in mean time to death in both generations.

### 3.4 Candidate genes

Prioritized genomic blocks on chromosome 25, defined as regions explaining more than 1% of the adjusted phenotypic variance for at least one BKD resistance trait, were further inspected for candidate genes. Based on this criterion, candidate genes were identified in Blocks 3, 4, 9, and 10 (Table 6).

**TABLE 6:** Candidate genes identified within prioritized block regions on chromosome 25 in *Salmo salar*.

Association region	Gene ID
Block 9 (31.7–32.0 Mb)	*fam46c*
	*man1a2*
	*trim45*
	*vtcn1*
Block 4 (24.36–24.45 Mb)	*pofut2*
Block 3 (22.07–22.29 Mb)	*stam2*
Block 10* (34.09 Mb)	*fstl1b*

*Block 10 contains a single SNP.

Block 9, located between 31.7 and 32.0 Mb, contained the largest number of prioritized candidate genes, including *fam46c*, *man1a2*, *trim45*, and *vtcn1*. Additional candidate genes were identified in Block 4, where *pofut2* was located, and in Block 3, which contained *stam2*. Block 10, represented by a single significant SNP, contained *fstl1b*. In total, seven candidate genes were identified within prioritized regions on chromosome 25.

These genes were selected as potential candidates associated with BKD resistance based on their location within genomic regions showing relevant phenotypic variance explained. The complete list of genes located within each block is provided in Supplementary Table 1.5. Among the SNPs listed in Table 4, AX-88118415 was the only variant located within a prioritised candidate gene, occurring in a non-coding region of *man1a2*. No additional Table 4 SNPs were located within the other selected candidate genes. Several SNPs were located within non-candidate genes, including AX-88110791 within *wdr3*.

## 4 Discussion

### 4.1 Disease challenge

BKD controlled challenges have been used for different objectives, such as performing GWAS to investigate host resistance (Holborn et al., 2018), investigating the influence of water temperature on the host immune response (Rozas-Serri et al., 2020), and identifying immunopathological biomarkers to define a robust resistance phenotype (Eslamloo et al., 2020).This resistance challenge was applied due to its ability to capture differential responses across multiple biological levels, ranging from individual fish to tank-level variation. In the present study, it enabled the characterization of both individual phenotypic responses and variation across generations.

Average weight is one of the variables that diverges most among BKD challenges, ranging from 25 g in Rozas-Serri et al. (2020) to 537 ± 119 g in Holborn et al. (2018). The present study utilized fish with mean weights of 54.8 g and 89.3 g, placing them in a similar life-stage category to the 54 ± 6 g parr used in Eslamloo et al. (2020). Both year classes were pre-smolts, although YC22 fish were older than YC18 fish (approximately 540 vs. 404 days), which likely contributed to differences in body size and growth. Despite these differences, we found no significant correlation between initial body weight and mortality. Across generations, YC22 showed greater weight gain than YC18 (32% vs. 16%). In addition to differences in age and initial body size, variation in feeding regimes between year classes may also have contributed to the observed differences in growth during the challenge. Importantly, the consistent chromosome 25 association observed in the year-class-specific GWAS suggests that the main genetic signal for BKD resistance was reproducible despite differences in age, body size, and experimental conditions between cohorts.

The differences in mortality and time to death averages could be partly attributed to variation in *R. salmoninarum* inoculum concentration, which was higher in YC22 ( $1.0\times 10^{9}$ CFU/mL) compared to YC18 ( $6.4\times 10^{8}$ CFU/mL), representing an increase of approximately 56%. This higher bacterial load may have influenced infection dynamics and contributed to the observed phenotypic variation. Additionally, although the same *Renibacterium salmoninarum* isolate and inoculation protocol were used in both trials, ensuring consistent virulence across independent challenge experiments is inherently difficult. Disease challenges are influenced by complex host–pathogen interactions and multiple environmental and biological factors, which can affect pathogen virulence and make it difficult to reproduce identical infection dynamics across trials (Kennedy et al., 2016).

### 4.2 Heritability

Reported heritability estimates for BKD resistance in Canadian Atlantic salmon were 0.143 for mortality and 0.145 for time to death when using genomic information (Holborn et al., 2018). In European populations, estimates based on pedigree information and different statistical models ranged from 0.23 ± 0.10 to 0.27 ± 0.08 (Gjedrem and Gjøen, 1995). In the present study, heritability estimates obtained using the full dataset were 0.14 ± 0.01 for mortality and 0.19 ± 0.01 for time to death, which are close to those reported for the Canadian population. When generations were analyzed separately, heritability estimates varied between YC18 and YC22. In YC18, estimates were 0.1474±0.0240 for mortality and 0.1846±0.0247 for time of death, whereas in YC22 they increased to 0.2198±0.0261 and 0.2971±0.0260 , respectively. The higher estimates observed in YC22 suggest that this generation captured a larger proportion of additive genetic variation for BKD resistance. This difference may be partly related to variation in challenge conditions between generations, as discussed previously, which could have increased phenotypic variability among individuals.

Overall, the estimates obtained in this study indicate a low-to-moderate additive genetic contribution to variation in BKD resistance traits. While the full-dataset estimates were similar to those reported by Holborn et al. (2018), the generation-specific estimates, particularly in YC22, were closer to the range reported by Gjedrem and Gjøen (1995).

### 4.3 Genome wide association study

The GWAS performed for both traits revealed a genetic architecture that differed from the pattern previously reported by Holborn et al. (2018), who found no strong genome-wide associations for either BKD resistance trait. In contrast, our analysis identified a region on chromosome 25 that has not been previously reported, containing a cluster of SNPs significantly associated with resistance to the pathogen infection. Importantly, the same relevant association signal on chromosome 25 was observed when the GWAS was performed independently for each year class, supporting the consistency of this region across both challenges (Supplementary Figure 1.1). The Manhattan plots suggest the presence of a region with a concentrated genetic signal; however, given the low phenotypic variance explained observed, the overall architecture is more consistent with a polygenic trait influenced by loci of moderate effect rather than an oligogenic model driven by a few major-effect regions. The polygenic architecture supported by this study has direct implications for breeding programs aiming to improve BKD resistance. Under this scenario, marker-assisted selection (MAS) based on a small number of significant SNPs would likely be inefficient, as these markers would capture only a limited proportion of the genetic variation underlying resistance. In contrast, genomic selection is expected to be a more effective approach, because it uses genome-wide marker information to capture the cumulative effects of multiple QTL with small to moderate effects that are in linkage disequilibrium with nearby SNPs.

In addition to survival related traits, post-infection growth maintenance may also represent a useful complementary phenotype for future genomic studies of BKD response. Although disease tolerance was not directly evaluated here because pathogen burden was not measured and weight change may have been influenced by mortality, differences in feeding and rearing conditions, and genetic variation in growth rate among individuals and families, future studies combining repeated growth measurements with pathogen load data could support GWAS aimed at characterizing the genomic architecture of BKD tolerance.

### 4.4 Haplotype analysis

The haplotype analysis provided additional insight into the chromosome 25 association signal detected in the GWAS. Rather than interpreting each significant SNP independently, the associated markers were evaluated within genomic blocks, allowing the regional structure of the signal to be considered. This is particularly relevant for complex traits such as disease resistance, where multiple linked markers may collectively capture the effect of one or more underlying QTL. By accounting for the correlation among nearby SNPs, this approach helps reduce the risk of interpreting isolated associations and provides a more biologically meaningful interpretation of the GWAS results (Duggal et al., 2008). The identification of 13 genomic blocks on chromosome 25 supports the presence of a structured regional signal rather than a single-marker effect. Among these regions, Block nine showed the strongest evidence of association, explaining 3.3% of the phenotypic variance and 16.8% of the genetic variance for the time to death trait, and 2.3% and 15.65% respectively for mortality. Although the phenotypic variance explained by this block was modest, its contribution to genetic variance suggests that this region may represent a relevant component of the genetic architecture underlying BKD resistance. This is consistent with the expected behavior of polygenic traits, in which individual regions generally explain limited phenotypic variance but may still contribute meaningfully to the additive genetic variation available for selection (Cambien, 2011). Within Block 9, the haplotype analysis further refined the association signal by identifying a favorable AA–CC haplotype group. Individuals carrying this haplotype showed improved resistance across both evaluated traits, with lower binary mortality and longer survival time under challenge conditions. The observed reduction in mortality of 17.45% and the increase of approximately 3 days in survival time suggest that this haplotype may be capturing a relevant local genetic effect. Importantly, the same favorable pattern was also observed within families, indicating that the association is unlikely to be explained only by between-family structure (results not shown). This within-family consistency strengthens the evidence that the haplotype may be linked to genetic variation affecting BKD resistance. From a breeding perspective, these findings suggest that the chromosome 25 region, and particularly the Block nine haplotype, may provide useful complementary information for selection. However, the relatively modest PVE and the broader polygenic architecture observed for BKD resistance indicate that this haplotype should not be used as a stand-alone marker-assisted selection criterion, even though individuals carrying the favorable genotype showed up to a 17.5% reduction in mortality. Instead, it may be more appropriately considered as a complementary marker within a genomic selection framework, provided that its effect is validated in additional generations and independent populations. Therefore, the AA–CC haplotype in Block nine represents a candidate regional signal that could help refine future selection decisions. These findings could also have practical applications in specific breeding scenarios. For example, after genomic selection has been applied at the nucleus level, this haplotype could be incorporated into a low-density SNP chip to pre-select more BKD-resistant candidates in populations chronically affected by the bacteria, providing a lower-cost complementary tool for downstream selection stages.

### 4.5 Biological relevance of candidate genes

Candidate genes were investigated within blocks exceeding 1% adjusted PVE for at least one trait (Blocks 3, 4, 9, and 10). This criterion was intended to prioritise regions for exploratory biological interpretation; the modest PVE values of individual blocks are consistent with the broader polygenic architecture of BKD resistance. To facilitate interpretation, genes were organized into functional categories based on their biological roles.

The first and most relevant category for BKD resistance comprises genes involved in immune regulation, as they directly modulate host responses to infection. This group is mainly composed of Block nine genes, including *vtcn1* and *trim45*, as well as *stam2* from Block 3. Together, these genes influence key immune signaling pathways and the regulation of inflammatory responses. A strong candidate based on its functional relevance is *vtcn1*, which has been described as a T cell inhibitor that downregulates immune responses, including suppression of pro-inflammatory functions (Vaishnav et al., 2022). Consistently, deficiency of *vtcn1* has been shown to enhance innate immunity against bacterial pathogens in mouse models, supporting its role as a negative regulator of immune activation (Zhu et al., 2009). Similarly, *stam2* has been reported as a negative regulator of innate immunity in fish. In Miiuy croaker infected with *Vibrio harveyi*, its suppression leads to increased bacterial proliferation, an effect mediated through inhibition of the NF- κ B signaling pathway and reduced production of pro-inflammatory cytokines (Xu et al., 2024). In addition, *trim45* has been proposed as a candidate gene for resistance to sea lice in *Salmo salar* based on GWAS analyses (Cáceres et al., 2021), and is involved in the regulation of inflammatory responses (Xia et al., 2022; Zhang et al., 2025).

A second category includes genes involved in cellular stress and homeostatic regulation. The gene *man1a2* is involved in cellular homeostasis and stress regulation. In mouse models, the deficiency of it activates stress pathways and promotes pathological inflammation through increased expression of inflammatory genes, highlighting its role in modulating immune responses relevant to disease resistance (Ningappa et al., 2021).

A third group includes genes related to immune effector functions and tissue regulation. The *fam46c*/*tent5c* genes are involved in immune function, particularly in regulating secretory capacity and immunoglobulin production in plasma cells (Lacidogna et al., 2025). Similarly, *fstl1b* encodes a glycoprotein associated with pro-inflammatory processes, macrophage regulation, and tissue homeostasis, highlighting its role in inflammatory control (Guggeri et al., 2024). Finally, *pofut2*, although not yet studied in fish, has been implicated in human cancer immunity, where elevated expression has been linked to increased proliferation, invasion, and angiogenic capacity of tumor cells, further supporting its role in immune-modulated disease processes (Zou et al., 2026).

Another interesting finding is the block six on chromosome 25 (28,251,241–28,510,871) partially overlapping the maturation-associated region reported by Sinclair-Waters et al. (2022) (28,389,273–28,889,273), which lies near the well-characterised *vgll3* maturation locus. Variation at this locus is strongly associated with sea age at maturity in Atlantic salmon (Ayllon et al., 2015). The *vgll3* region may influence energy allocation and metabolic conditions, which could indirectly affect the response to infection. Previous studies have associated this region with differences in lipid storage and utilization in juvenile Atlantic salmon, including immature fish (House et al., 2025). However, this proposed relationship remains hypothetical and has not been experimentally validated in the populations evaluated in the present study.

These candidate genes suggest a potential role of haplotypic variability in BKD resistance, particularly through pathways related to inflammation and bacterial proliferation. However, further validation at the molecular level is required to confirm their functional relevance, for example, through transcriptomic analyses as performed by Eslamloo et al. (2020), who applied similar methodologies in a disease challenge context. In that study, as in the present interpretation, T-cell activation and differentiation were identified as critical processes associated with candidate genes. Specifically, Eslamloo et al. (2020) validated several T-cell markers, including *rnf144a* and *tnfrsf6b*, highlighting the importance of adaptive immune regulation in the response to disease. Similarly, Rozas-Serri et al. (2020) performed transcriptomic analyses of BKD infection in *S. salar* and found that highly infected fish exhibited a marked downregulation of key T-cell markers. This transcriptomic evidence of T-cell suppression is further supported by genomic association studies that identified the V-set domain containing T cell activation inhibitor 1 as a primary candidate gene for BKD resistance. Located within a significant genomic block (Block 9) on Chromosome 25, the presence of this known negative regulator of T-cell responses provides a potential genetic basis for the observed failure to mount an effective cell-mediated immune response in susceptible individuals. However, this proposed mechanism was not directly evaluated in the present study, as cell-mediated immune responses were not measured in the two salmon populations used for the GWAS, and therefore requires further validation.

## 5 Conclusion

This study characterized the genetic architecture of resistance to Bacterial Kidney Disease (BKD) in Atlantic salmon across two generations. Heritability estimates of 0.14 for mortality and 0.19 for time to death confirmed the presence of additive genetic variation for BKD resistance, indicating that this trait can be improved through selective breeding. Genome-wide association analyses identified a genomic region on chromosome 25 significantly associated with both mortality and time-to-death traits. This region showed consistent associations across generations and explained a moderate proportion of the additive genetic variance, supporting the conclusion that BKD resistance is influenced by loci of moderate effect within a broader polygenic architecture. Haplotype analysis within the chromosome 25 region identified favorable genotypes associated with reduced mortality and increased survival time under challenge conditions. Further validation in additional independent populations will be required before implementation in breeding programs, supporting the development of sustainable strategies aiming for disease resistance in Atlantic salmon.

## Data Availability

The datasets presented in this study can be found in online repositories. The names of the repository/repositories and accession number(s) can be found in the article/Supplementary Material.
