# Supplementary material for: Genome-wide association study and haplotype characterization of bacterial kidney disease (BKD) resistance in Atlantic salmon (Salmo salar) across two generations
Source: Front Genet. 2026 Oct 28;17:1985591. doi: 10.3389/fgene.2026.1985591 (PMC13752854; doi:10.3389/fgene.2026.1985591)
Supplement: Supplementary file 1 [file DataSheet1.pdf]

Supplementary Material

1 SUPPLEMENTARY TABLES AND FIGURES

1.1 GWAS for each generation

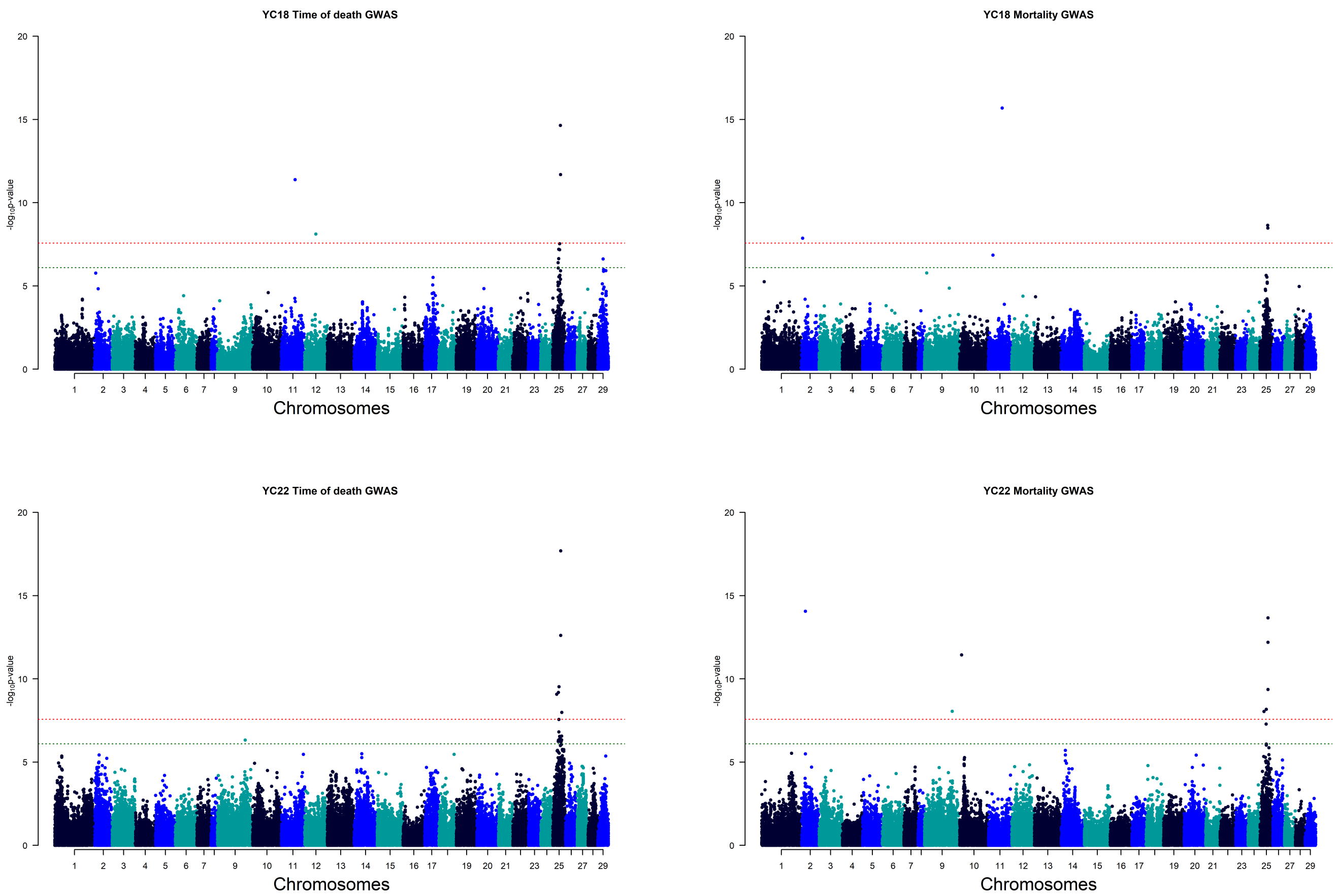

Figure S1: Supplementary Figure S1. Manhattan plots for BKD time and BKD mortality in YC18 (top row) and YC22 (bottom row).

1.2 QQ plots for GWAS

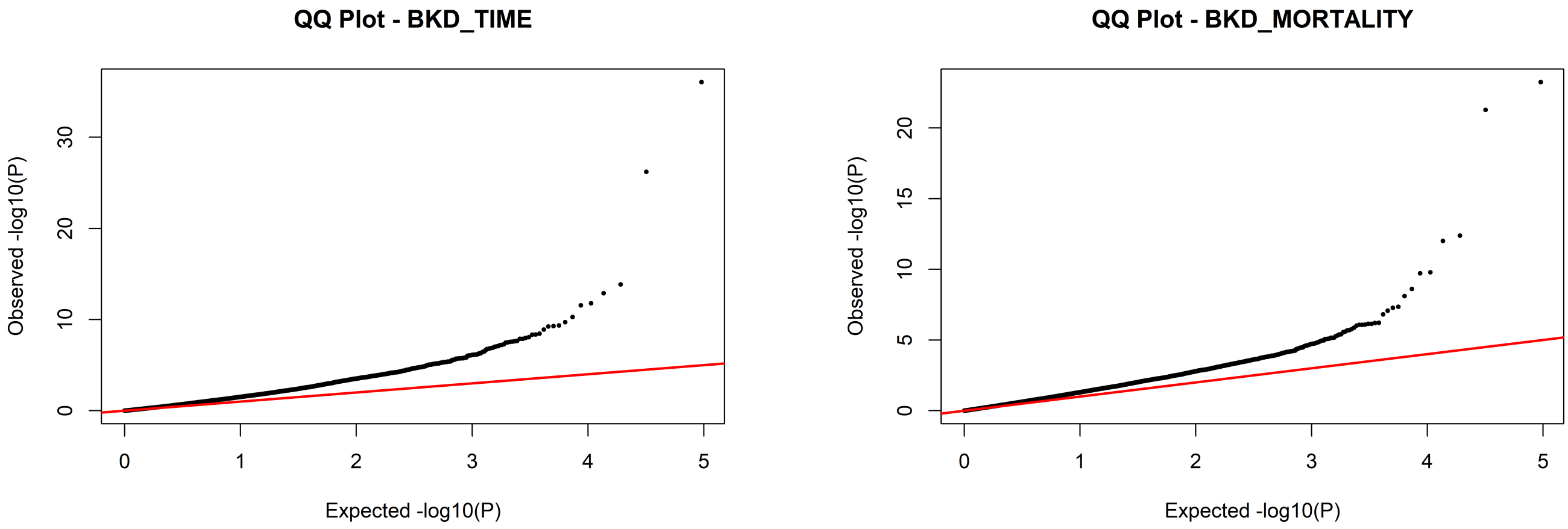

Figure S2: QQ plots of GWAS p-values for Time of Death (left) and Mortality (right) traits.

1.3 All significant SNPs

Table S1: Top SNPs associated with time-to-event and mortality. Ordered by significance for Time.

Chr	SNP	bp	MAF	b (Time)	p (Time)	p (Mortality)
25	AX-88110791	31728863	0.438	-2.22296	5.94e-36	5.75e-24
25	AX-88118415	31824753	0.379	-1.94511	6.37e-26	5.27e-22
25	AX-87164891	32044678	0.483	1.35423	1.07e-13	9.96e-13
25	AX-98296355	25528053	0.310	1.51321	2.22e-13	2.45e-09
11	AX-87473182	55575129	0.147	1.61941	1.12e-09	4.18e-13
25	AX-96172802	24359232	0.456	1.27498	2.21e-12	1.66e-10
25	AX-96426425	22289341	0.353	-1.27697	3.12e-12	1.95e-10
25	AX-88300717	29567889	0.421	-1.19568	1.09e-10	-
9	AX-87691295	111819571	0.204	1.46256	1.94e-10	8.50e-07
22	AX-87432240	32568592	0.153	-1.62343	3.79e-10	-
25	AX-96134336	37772413	0.103	-1.77415	1.78e-09	-
25	AX-96352799	31748699	0.213	1.28980	2.30e-09	4.49e-08
25	AX-88213524	38944413	0.246	1.21221	2.89e-09	7.28e-07
25	AX-98309383	38950285	0.244	1.21705	3.90e-09	-
25	AX-96130927	25027279	0.295	1.13746	6.20e-09	8.00e-09
25	AX-96458203	22076103	0.310	1.20123	7.37e-09	1.57e-07
25	AX-96497525	24450130	0.220	-1.21220	1.07e-08	6.38e-07
25	AX-96336032	24569982	0.198	-1.26119	1.23e-08	-
14	AX-87544078	53485361	0.416	1.01643	1.70e-08	-
25	AX-87465426	24396219	0.289	-1.14838	2.00e-08	8.45e-08

Continued on next page

Table S1 – continued from previous page

Chr	SNP	bp	MAF	b (Time)	p (Time)	p (Mortality)
2	AX-88147225	17417340	0.349	-	-	5.29e-08
17	AX-97887738	30525905	0.166	-1.38023	5.73e-08	-
25	AX-96179822	31707394	0.134	1.40656	5.89e-08	8.29e-07
22	AX-96163601	61041163	0.419	-0.99930	6.19e-08	-
25	AX-87839598	22211312	0.351	-1.01414	6.19e-08	-
25	AX-96501235	34086405	0.400	-1.02212	7.89e-08	-
25	AX-88302520	29454134	0.132	1.38485	1.00e-07	-
25	AX-88033143	28510871	0.241	-1.14610	1.04e-07	-
25	AX-87763914	21503369	0.132	-1.38893	1.18e-07	-
25	AX-96360593	41586870	0.227	1.13290	1.33e-07	-
25	AX-98303652	30608135	0.292	-1.07372	1.57e-07	-
29	AX-96221996	25066709	0.318	1.02333	1.98e-07	-
29	AX-87825217	22736968	0.437	0.95988	2.02e-07	-
25	AX-96197985	18226525	0.288	-1.06099	2.03e-07	-
14	AX-87294504	53778163	0.321	-0.97144	2.50e-07	-
25	AX-87502204	29535756	0.283	-1.04556	2.69e-07	-
25	AX-96503479	37834492	0.491	0.94688	2.82e-07	-
14	AX-87406572	53853090	0.321	-0.96519	3.04e-07	-
29	AX-87596907	22724881	0.439	0.94121	3.91e-07	-
25	AX-96140213	39029963	0.355	0.94063	4.04e-07	-
25	AX-96460974	28251241	0.205	-1.18833	4.15e-07	-
17	AX-87230881	37638429	0.134	-1.41149	4.35e-07	-
17	AX-96483550	35856440	0.348	-0.98082	4.36e-07	-
25	AX-87827613	28251655	0.203	-1.19940	4.59e-07	-
25	AX-417119873	29709421	0.335	-0.91793	5.46e-07	-
17	AX-96120243	35248090	0.302	-0.99302	5.56e-07	-
2	AX-417104234	6270950	0.106	-	-	6.16e-07
25	AX-87511379	38167816	0.462	0.93471	6.76e-07	-
25	AX-96293577	38951774	0.440	-	-	7.12e-07
25	AX-96439263	30609053	0.261	-1.03240	9.11e-07	-
25	AX-87482234	39112658	0.498	-0.85081	9.50e-07	-
25	AX-96352807	31562357	0.104	-	-	9.70e-07
10	AX-87482847	18185378	0.447	-	-	8.49e-07
12	AX-87455402	46037519	0.170	1.20696	9.90e-07	-

1.4 SNP Combination Summary Table

Table S2. Summary of SNP combinations with mean mortality, mean time in days, and sample size, ordered by mean mortality.

SNP combo	Mean Mortality	Mean Time (days)	n
AA-CC-CC	55.0000	52.1665	91
AA-CC-TT	62.3708	51.5264	782
AA-CC-TC	66.5164	50.5784	519
CA-CC-TT	72.6974	49.0970	195
CA-CC-TC	73.7778	47.5331	99
CA-TC-TC	74.1164	49.1968	1374
CA-TC-CC	76.3442	48.7080	491
CA-TC-TT	78.4318	48.7582	88
CC-TT-CC	81.7231	47.4988	567
CC-TC-CC	82.3000	47.3399	80
CC-TC-TC	83.7872	45.7446	141
CC-TT-TC	89.4681	45.4698	47
CC-TC-TT	100.0000	40.8300	6

1.5 Genes within genomic blocks

Table S3. Candidate genes identified across genomic blocks. Selected candidate genes are shown in bold.

Block	Gene Symbol	Start	End	Gene Name
Block 9	tbx15	31564606	31587437	T-box transcription factor 15
	LOC106586552	31665589	31667253	zona pellucida-like domain-containing protein 1
	LOC106586553	31676473	31678840	zona pellucida-like domain-containing protein 1
	LOC106586554	31728332	31742531	WD repeat-containing protein 3
	LOC106586605	31742316	31776510	ganglioside-induced differentiation-associated protein 2
	tent5c	31782294	31788411	terminal nucleotidyltransferase 5C
	**LOC106586607**	**31814665**	**31942477**	**mannosidase, alpha, class 1A, member 2**
	**LOC106586608**	**31950326**	**31955119**	**tripartite motif-containing protein 45**
	**LOC106586567**	**31955358**	**31958123**	**V-set domain containing T cell activation inhibitor 1**
	LOC106586555	31988163	31994019	uncharacterized LOC106586555
	LOC123730538	32029677	32032887	uncharacterized LOC123730538
Block 4	LOC106586437	24400406	24422061	basic leucine zipper and W2 domain-containing protein 1-A
	LOC106586438	24428280	24438466	trans-1,2-dihydrobenzene-1,2-diol dehydrogenase
	LOC106586439	24439451	24582132	unconventional myosin-Ib
	**pofut2**	**24585974**	**24602371**	**protein O-fucosyltransferase 2**
	abca12	24612608	24707027	ATP-binding cassette, sub-family A (ABC1), member 12
	LOC106586442	24723978	24727618	uncharacterized LOC106586442
	LOC106586443	24741098	24751099	BRCA1-associated RING domain protein 1
	LOC123730557	24773548	24777001	BRCA1-associated RING domain protein 1-like
	vwc2l	24777900	24804363	von Willebrand factor C domain containing 2 like
Block 3	neb	22022107	22101571	nebulin
	arl5a	22114942	22134504	ADP-ribosylation factor-like 5A
	LOC106586393	22135083	22188608	voltage-dependent L-type calcium channel subunit beta-4
	LOC106599760	22189546	22189790	uncharacterized LOC106599760
	**LOC106586394**	**22190600**	**22206362**	**signal transducing adapter molecule 2**
	phs1	22206763	22214636	PHS1 protein
	LOC106586396	22219339	22359828	adenylate cyclase type 5
Block 10	LOC106586642	34028544	34036997	nectin-3
	LOC106586572	34049192	34059874	uncharacterized LOC106586572
	LOC106586647	34072175	34099470	uncharacterized LOC106586647
	**LOC100136473**	**34116624**	**34158712**	**follistatin-related protein 1**

2 DATABASE

Phenotypic and genotypic data are available in Figshare: https://doi.org/10.6084/m9.figshare.32555262.

Researchers using this dataset in subsequent studies are requested to notify the author and ensure appropriate acknowledgment and attribution in any resulting publications, presentations, reports, or other scholarly outputs.
